# Narrow Escapes

**Published:** 1875-05

**Authors:** L. Alexander

**Affiliations:** Yorkville, S. C.


### NARROW ESCAPES.

By L. ALEXANDER, M.D., of Yorkville, S. C.

On the night of the 28th I was requested to go about seven miles in the country, to see a little girl, five years of age, whom the messenger supposed to be fatally injured from an accident about dark. Upon my arrival I was informed that the child, while standing between the front and rear seats of a heavily loaded buggy-wagon, had fallen between the wheels, her head first striking the ground, and bearing the entire weight of the hind wheel of this heavily-loaded vehicle. Examination showed a lacerated wound of the scalp, immediately above the left ear, without fracture of the skull or any further damage. After receiving proper attention she became quite cheerful, and the following morning was apparently as well and happy as ever.

This case is not so remarkable within itself, but shows how frequently accidents, befalling children, which we might suppose would result fatally, do comparatively little harm, and calls to my mind several similar cases, two in particular, in which nature seems to have lent a helping hand.

The first occurred several years ago, while I was practicing in Virginia, and is that of a little child, just beginning to walk, who crawled upon the railroad track just in time to meet the express train running at full speed. The little fellow was struck by the cow-catcher, and knocked about fourteen feet, only inflicting some slight cuts and scratches upon the head, where it came in contact with the ground, and causing a copious discharge of fæcal matter. Not the slightest bruise was found upon the body.

The second case occurred some months since, in which a boy, eight years of age, fell from a large wagon, heavily loaded with green, oak boards, and had both the front and hind wheels to pass diagonally across his body, leaving only a slight bruise (which soon disappeared) to indicate the passage of the wheels.

